# Anticipating the future: prognostic tools as a complementary strategy to improve care for patients with febrile illnesses in resource-limited settings

**DOI:** 10.1136/bmjgh-2021-006057

**Published:** 2021-07-30

**Authors:** Arjun Chandna, Jennifer Osborn, Quique Bassat, David Bell, Sakib Burza, Valérie D’Acremont, B Leticia Fernandez-Carballo, Kevin C Kain, Mayfong Mayxay, Matthew Wiens, Sabine Dittrich

**Affiliations:** 1 Cambodia Oxford Medical Research Unit, Angkor Hospital for Children, Siem Reap, Cambodia; 2 Centre for Tropical Medicine & Global Health, University of Oxford, Oxford, UK; 3 Foundation for Innovative New Diagnostics, Geneva, Switzerland; 4 ISGlobal, Hospital Clínic, Universitat de Barcelona, Barcelona, Spain; 5 Centro de Investigação em Saúde de Manhiça (CISM), Maputo, Mozambique; 6 ICREA, Pg. Lluís Companys 23, Barcelona, Spain; 7 Pediatrics Department, Hospital Sant Joan de Dé, Universitat de Barcelona, Esplugues, Barcelona, Spain; 8 Consorcio de Investigación Biomédica en Red de Epidemiología y Salud Pública (CIBERESP), Madrid, Spain; 9 Independent Consultant, Issaquah, Washington, USA; 10 Médecins Sans Frontières, New Delhi, India; 11 Centre for Primary Care and Public Health, University of Lausanne, Lausanne, Switzerland; 12 Swiss Tropical and Public Health Institute, Basel, Switzerland; 13 Department of Medicine, University Health Network, Toronto, Ontario, Canada; 14 Microbiology Department, Lao-Oxford-Mahosot Hospital-Wellcome Trust Research Unit, Vientiane, Lao People's Democratic Republic; 15 Institute of Research and Education Development (IRED), University of Health Sciences, Vientiane, Lao People's Democratic Republic; 16 Center for International Child Health, BC Children's Hospital, Vancouver, British Columbia, Canada; 17 Mbarara University of Science and Technology, Mbarara, Uganda; 18 Department of Anesthesiology, Pharmacology & Therapeutics, University of British Columbia, Vancouver, British Columbia, Canada; 19 Walimu, Kampala, Uganda

**Keywords:** infections, diseases, disorders, injuries, diagnostics and tools, health systems

## Abstract

In low-income and middle-income countries, most patients with febrile illnesses present to peripheral levels of the health system where diagnostic capacity is very limited. In these contexts, accurate risk stratification can be particularly impactful, helping to guide allocation of scarce resources to ensure timely and tailored care. However, reporting of prognostic research is often imprecise and few prognostic tests or algorithms are translated into clinical practice.

Here, we review the often-conflated concepts of prognosis and diagnosis, with a focus on patients with febrile illnesses. Drawing on a recent global stakeholder consultation, we apply these concepts to propose three use-cases for prognostic tools in the management of febrile illnesses in resource-limited settings: (1) guiding referrals from the community to higher-level care; (2) informing resource allocation for patients admitted to hospital and (3) identifying patients who may benefit from closer follow-up post-hospital discharge. We explore the practical implications for new technologies and reflect on the challenges and knowledge gaps that must be addressed before this approach could be incorporated into routine care settings.

Our intention is that these use-cases, alongside other recent initiatives, will help to promote a harmonised yet contextualised approach for prognostic research in febrile illness. We argue that this is especially important given the heterogeneous settings in which care is often provided for patients with febrile illnesses living in low-income and middle-income countries.

**Summary box**

- Prognostic tools can improve the efficiency and utility of management algorithms for patients with febrile illnesses; a recent multinational stakeholder consultation identified that they could be particularly impactful in settings where diagnostic capacity is most limited.
- Clearly defined use-cases can help to focus efforts of researchers, product developers and policy makers to ensure that the proposed solutions are appropriate and relevant for the targeted contexts.
- Novel prognostic tools should improve recognition of impending serious illness in patients who lack clinical signs of severity as determined by existing algorithms, be subject to robust cost–benefit assessments, and be developed in partnership with end-users to ensure they function within the limited human and material resources available at the peripheral levels of most low-income and middle-income country health systems
- Guidance to standardise measurement of candidate predictors and harmonise outcome assessments has recently been developed and should be used to contextualise results, facilitate data sharing and maximise comparability of findings from disparate studies

## Introduction

Globally, febrile illnesses are among the most common reasons to seek healthcare.^1^ While most can be managed at the community level, a small proportion (~1%–2%) progress to life-threatening disease.^2^ This burden is carried disproportionately by individuals in low-income and middle-income countries (LMICs), where febrile illnesses remain a leading cause of morbidity.^3^

Understanding the underlying causes of the main febrile syndromes is critical to successful treatment of febrile illnesses. Several recent initiatives have addressed this topic.^4–6^ Nevertheless, approaches that focus solely on diagnosis struggle to reconcile the fact that patients with the same infection or syndrome can have markedly different illness trajectories,^7^ perhaps reflecting differing host nutritional and other susceptibility states.

Most febrile patients in LMICs are managed by community health workers and healthcare providers working at primary or district level. These practitioners often have limited training and inadequate access to the necessary supervision and diagnostic testing to support their clinical decision-making. In such contexts, in addition to assessing the cause of a patient’s illness, an equally pertinent question is: *is my patient’s condition likely to progress and require a higher level of care?* A prognostic tool that could reliably risk stratify patients would have immense potential for benefit, through timely identification of patients at risk of deterioration and guiding appropriate use of scarce resources.

In contrast to a *diagnostic* test which determines whether a specific disease or health state is present at the moment the test is performed, a *prognostic* test provides information on the likelihood of a particular outcome occurring in the future.^8^ Used appropriately, prognosis can complement diagnosis to improve precision and efficiency of management algorithms for febrile illnesses. This could be particularly impactful in resource-constrained settings where diagnosis remains most challenging, triaging practices predominantly rely on clinical evaluation, and decisions to refer must be made early due to complex context-related referral mechanisms.

Common pathophysiological pathways to severe febrile illness exist across a range of microbial aetiologies.^9 10^ Biochemical markers of these pathways, reflecting endothelial injury, immune activation and coagulation, appear to add value to simple bedside assessments to improve identification of patients with a poor prognosis.^11–13^ Reliable and practicable tests for these markers could help risk stratify febrile patients and inform management decisions at critical junctures in the patient care pathway. While a standalone test for a biochemical biomarker could provide useful prognostic information, these tests might be more effective as part of an algorithm, combining measurement of a biomarker(s) with other clinical parameters (signs and symptoms, demographic information, comorbidities, etc) to more accurately assess risk and guide rational management.

Unlike diagnosis, prognosis is inherently context-dependent: a patient’s eventual outcome is inextricably influenced by the available resources and quality of care. Hence, in order to advance the conversation around prognostic testing in febrile illnesses, specific use-cases must be defined. Each use-case should detail the clinical problem and consider the resources available to treat febrile illnesses in that setting (eg, health worker and laboratory capabilities, referral capacity, and availability of essential resources such as oxygen, fluids, antimicrobials and provision of vital organ support), in order to contextualise the outcomes against which a candidate prognostic test or algorithm is to be assessed.

In this paper, we first review the concepts of prognosis and diagnosis, with a focus on assessment of the severity of febrile illness. We then apply these concepts to define three potential use-cases for prognostic tools in the management of febrile illnesses in resource-limited settings: (1) guiding referrals from the community to higher-level care; (2) informing resource allocation for patients admitted to hospital and (3) identifying patients who may benefit from closer follow-up post-hospital discharge. For each use-case, we explore practical implications for new technologies, with an emphasis on the requirements for putative tests to measure biochemical biomarkers within various healthcare settings in LMICs. We conclude by reflecting on the challenges and knowledge gaps that must be addressed before prognostic tools could be incorporated into routine care settings, drawing on the findings from multiple recent stakeholder consultations.^14^

## Prognosis and diagnosis in the assessment of disease severity

Healthcare providers regularly integrate multiple sources of data (eg, patient demographics, comorbidities, clinical signs and symptoms, and results of radiological and laboratory investigations) to determine the ‘true’ underlying disease or health state of their patient. Depending on the temporal relationship between these baseline data (predictors) and the disease or health state in question (the outcome), these predictions are either diagnostic or prognostic.^8^

**Figure 1:**
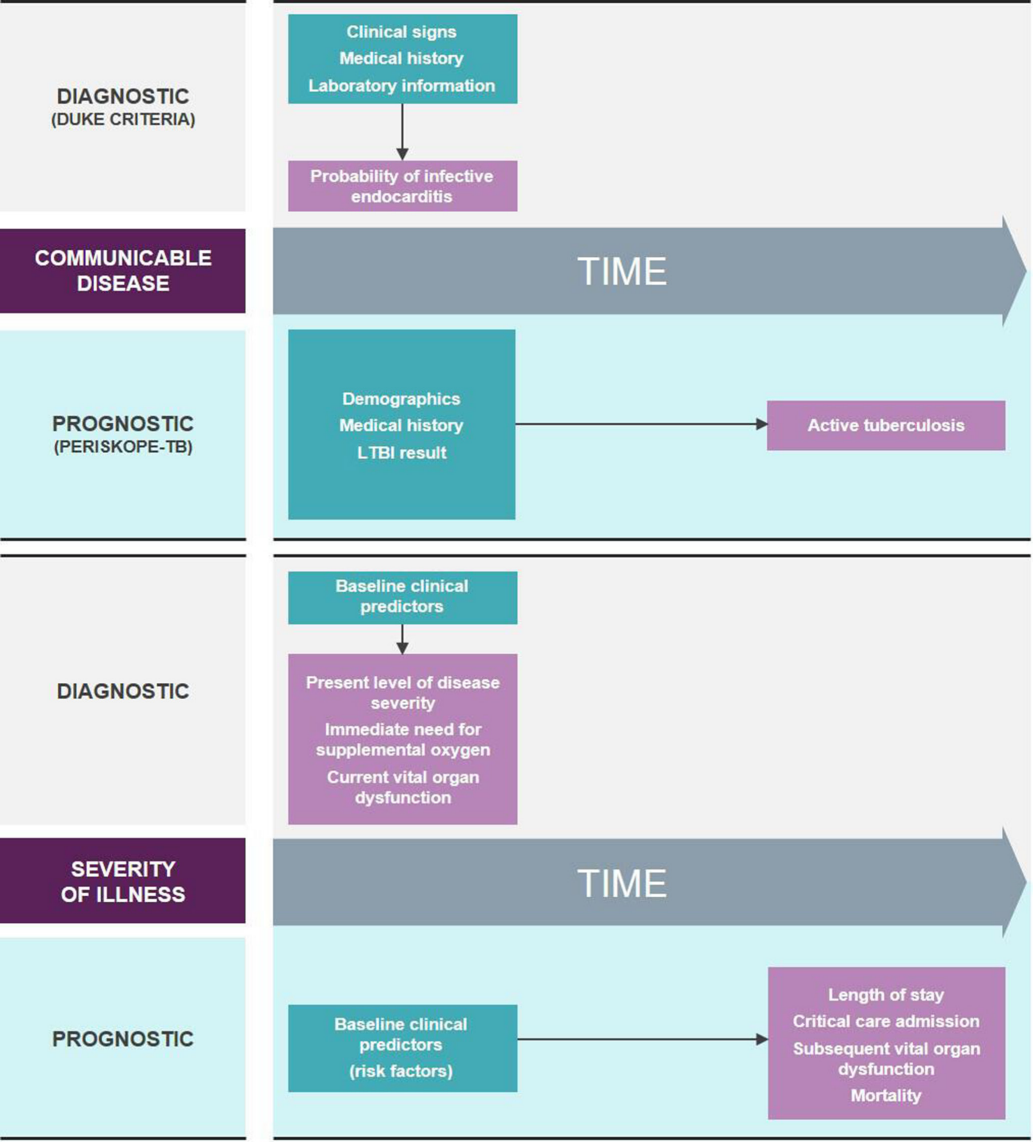
Classical paradigm for diagnostic and prognostic algorithms applied to communicable diseases (top) and the assessment of disease severity (bottom). Green boxes contain examples of baseline data (predictors) and pink boxes contain examples of diseases or health states (outcomes). Thin arrows indicate temporal relationship between predictors and outcomes. LTBI, latent tuberculosis infection; PERISKOPE-TB, personalized risk predictor for incident TB.

Often the distinction between diagnosis and prognosis is clear: integration of clinical, laboratory and radiological information to predict whether a patient may have infective endocarditis (Duke criteria)^15^ is a *diagnostic* process, whereas predicting the probability that an individual may develop active tuberculosis (TB) within the next 2 years based on their demographics, medical history and latent TB infection test result (PERISKOPE-TB)^16^ is easily recognisable as *prognosis* (figure 1).

For predictors of severity these concepts can become blurred. A patient’s severity reflects their likelihood of a poor outcome and hence predictors of severity are inherently prognostic. However, certain predictors also indicate a patient’s ‘level of severity’ at the time of measurement (eg, peripheral oxygen saturation in a patient with pneumonia) and in this sense, as well as providing prognostic information, can also be considered diagnostic of a patient’s severity at that moment. This is in contrast to other predictors that are primarily harbingers of future deterioration in patients who appear otherwise well (eg, various clinical and laboratory parameters measured during the febrile phase of a dengue or COVID-19 infection).^7 17 18^

Most predictors used for the assessment of severity fall into the first group, serving both diagnostic and prognostic purposes. For example, many guidelines and tools devised to inform the management of febrile illnesses in resource-limited settings use ‘Danger Signs’ to identify patients who are severely ill at the time of assessment and at high risk of mortality.^19 20^ Hence, these ‘Danger Signs’ can be considered both diagnostic (of the severity of illness at the time of assessment) and prognostic (for future risk of death). However, their lack of sensitivity and specificity, and high interobserver variability, make their performance poor for these particular purposes.^21 22^ Improving identification of impending serious illness in patients that lack clinical signs of severity as determined by existing management algorithms is a global public health priority.^14^

**Figure 2:**
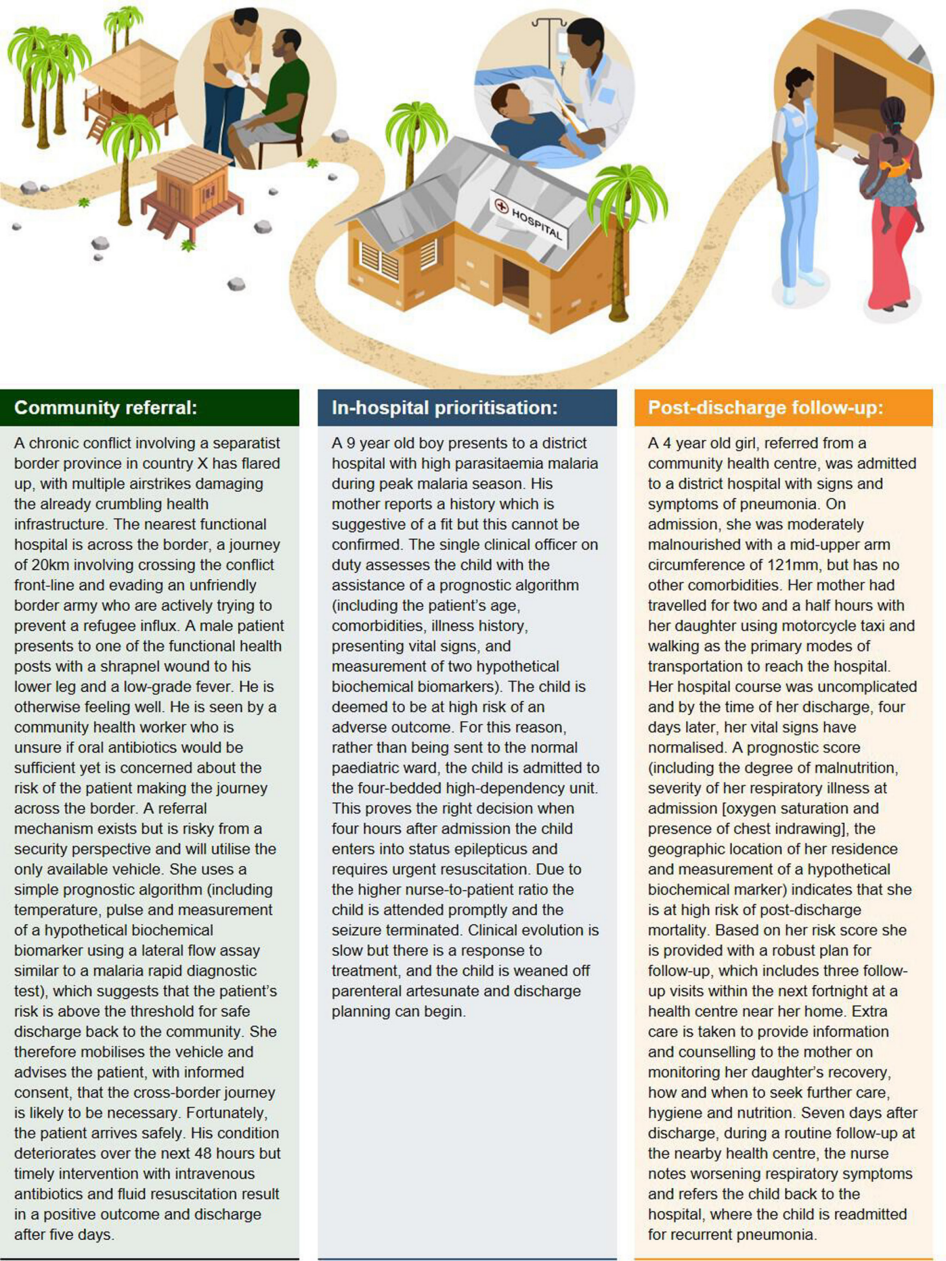
Clinical vignettes illustrating three use-cases for hypothetical prognostic tools in the management of febrile illnesses in resource-limited settings.

It is often poorly reported whether (and what proportion of) patients had met the predefined severity endpoint (eg, hospital admission, vital organ dysfunction or disease-specific severity scores) at the time the baseline predictors were measured (ie, whether the predictors are serving predominantly diagnostic or prognostic functions).^23–25^ This is particularly important in community settings where mortality is rare and may not be a feasible or relevant endpoint. Failure to identify a study as prognostic or diagnostic is a common shortcoming in the reporting of clinical prediction research.^26^ Recent guidance on the design, reporting and assessment of prognostic studies aims to improve this.^27^ To best leverage this, clearly defined use-cases for prognostic tools in the management of febrile illnesses (table 1; figure 2) are required to standardise data collection, encourage consistency of reporting, contextualise interpretation of results and maximise comparability of findings from disparate studies.

**Table 1:** Use-cases for prognostic tools in the management of febrile illnesses in resource-limited settings

Use-case	Healthcare context	Typical human and technical capacity for the management of febrile illnesses*	Relevant outcomes to assess candidate prognostic factors
(1) Referral or admission to hospital or maximal pre-referral care if referral not feasible.	Community health worker or village health volunteer in a rural village	Health workers are often lay people with a few days to months training and intermittent supervision by staff from the primary health centre or other actors implementing community-based healthcare programmes. A very limited range of equipment (eg, MUAC tapes, thermometers, respiratory rate counters), diagnostics (qualitative RDTs for malaria) and treatments (antipyretics, oral antibiotics or antimalarials, oral rehydration solution and nutritional supplements) may be available.	Persistence or worsening of symptoms; referral to hospital; admission to hospital
	Healthcare provider at primary health centre	Primary healthcare providers typically include clinical officers, nurses or midwives with a few months to years training. A greater range of clinical equipment (eg, pulse oximeters, weighing scales, stethoscopes) and diagnostic tests (eg, RDTs for other diseases and basic haematology) may be available. Some facilities may have the capacity for overnight observation and the delivery of intravenous fluids, antibiotics or nebulisers.	Persistence or worsening of symptoms; referral to hospital; admission to hospital.
	Healthcare provider in district hospital outpatient department	Healthcare staff can range from clinical officers with a few years training to experienced physicians. Similar clinical equipment available as at a primary health centre. Laboratory tests can also include instrumented platforms (which may be batched, depending on patient throughput). Proximity to inpatient care areas means threshold for admission for observation, further investigation and inpatient treatment may be lower.	Admission to hospital; length of hospital stay; admission to high-dependency area; measures of vital organ dysfunction.
(2) Prioritisation of human and material resources for hospitalised patients including admission to restricted-capacity high dependency areas and transfers to higher-level care.	Healthcare provider in district hospital inpatient department	Healthcare staff can range from clinical officers with a few years training to experienced physicians. A range of clinical equipment is available, as well as variable access to radiological (eg, point-of-care ultrasound), microbiological and laboratory testing. Frequent vital observations and delivery of supplemental oxygen therapy, intravenous medications and surgical interventions for source control may be possible. Admission of patients also permits evaluation of trends in clinical or laboratory parameters over time and response to therapeutic interventions to be observed.	Length of hospital stay; admission to high-dependency area; measures of vital organ dysfunction; mortality.
	Physician at admission to high dependency area or critical care unit in regional or tertiary hospital	Experienced physicians with access to clinical equipment and radiological, microbiological and laboratory testing. Near-patient tests such as blood gas machines and point-of-care ultrasound may be available in some settings, as may continuous vital sign monitoring and vital organ support (eg, inotropic therapy and non-invasive or mechanical ventilation).	Length of stay in high-dependency area; length of hospital stay; measures of vital organ dysfunction; mortality.
(3) Prioritisation for peri-discharge and post-discharge care interventions	Healthcare provider at hospital discharge	Range of healthcare staff, clinical equipment and radiological, microbiological and laboratory testing depending on the level of the health facility. Feasible to compare discharge measurements to those taken during the hospital stay (ability to look at trends over time and response to treatment). Some facilities may have access to community outreach teams or links with nearby community health facilities to assist with patient follow-up after discharge.	Readmission to a health facility; return to baseline health status; acute sequelae resolution; neurocognitive outcomes; mortality.

*Human and technical capacity varies greatly within countries and across regions: the examples given are for illustrative purposes and will not reflect all settings.

MUAC, mid-upper arm circumference; RDT, rapid diagnostic test.

## Use-cases for prognostic tools in the management of febrile illnesses in resource-limited settings

### Referral for higher-level medical care by community healthcare providers

Most patients with febrile illnesses present to peripheral levels of the health system.^1^ Distinguishing those that require referral can be difficult, and once identified the decision to transfer may not be straightforward. Particularly in rural areas and conflict settings, poorly functioning infrastructure, as well as geographic, climatic, social and political challenges mean that referral decisions often involve complex mechanisms and incur costs and risks for both patient and provider.^28^

Even with optimal deployment of existing algorithms, cases of serious illness can be missed and patients are inappropriately referred.^21^ In many settings these algorithms are not regularly used or are improperly applied due to various constraints common in LMICs.^22 29^ A prognostic test that could give community healthcare providers increased confidence in their decision to refer (or not) would have great potential to improve appropriateness of referrals and reduce resource misallocation.^30^ Increased confidence may also lead to better communication between providers and patients, which is important in contexts where strong traditional beliefs about causes and treatments of febrile illness exist.^31^

In settings where referral is not immediately feasible, accurate prognosis could guide provision of prereferral care, such as the first dose of parenteral antibiotics for suspected serious bacterial infections.^32^ During epidemics, like the current COVID-19 pandemic, identifying patients suitable for home-based management could prevent overburdening of health facilities. Such a prognostic test or algorithm would need to function with the limited human and material resources available at the peripheral levels of most LMIC health systems, and the threshold for referral adjusted according to the risks and benefits present in particular contexts, reflecting whether a high negative (NPV) or positive predictive value (PPV) is the priority.

Rather than being mutually exclusive, prognostic and diagnostic tests should be considered complementary: an algorithm integrating prognostic and diagnostic components can be envisioned as being highly useful in this context. First the algorithm could identify patients likely to benefit from referral for higher-level care, as described above, and second it could guide the further management of individuals who were identified as suitable for care at the community level (for example, informing appropriate antimicrobial prescription).

### Resource allocation for patients admitted to hospital

In many LMICs febrile illnesses remain the leading cause of hospitalisation.^33^ Particularly during seasonal outbreaks (eg, due to malaria, acute bacterial meningitis or dengue), health facilities are vulnerable to overcrowding and limited resources stretched further.^34^

Being able to predict the likely course of a patient’s illness given the resources available at a typical district-level hospital could enable better resource prioritisation—from simple measures such as facilitating early discharge or increased frequency of vital observations, to admission to restricted-capacity high dependency areas or informing timely transfer for tertiary-level care.

At regional-level and tertiary-level hospitals, accurate risk stratification might help direct resources towards patients more likely to benefit, for example, early institution of high-cost therapies and adjunctive procedures. This may reduce the likelihood of prolonged admission and subsequent long-term morbidity, and the financial burden of this on patients and their families.

### Identification of patients requiring closer follow-up after discharge from hospital

Survivors of severe infections are at increased risk of morbidity and mortality but this risk is modifiable with post-discharge care.^35 36^ However, outpatient follow-up and safety-netting is typically very difficult in LMICs and in conflict settings poor mobile phone coverage and internet blackouts pose additional challenges. A systematic review found that paediatric post-discharge mortality rates are often as high as those occurring in-hospital.^37^

Risk stratification of patients using data collected in the lead up to discharge would enable limited resources to be focused on more comprehensive follow-up of individuals at highest risk of post-discharge complications. Appropriate risk thresholds could be determined based on resources available for such a programme. Prognostic factors and algorithms that predict poor outcome following hospitalisation have been identified.^13 38^ Operationalising these for routine use would enable better targeting of peri-discharge and postdischarge interventions.^39^

### Prognostic tools in the context of clinical research and quality improvement initiatives

Prognostic tools could also improve management of febrile illnesses indirectly. Stratifying participant recruitment into trials of novel therapeutics by expected prognosis would ensure comparability between different sites, as well as selection of a study population in whom the attack rate is sufficiently high to adequately power the trial. Furthermore, if prognostic utility is verified, surrogate endpoints based on these markers could reduce the number of participants required, increasing the feasibility of conducting trials in more peripheral settings, allowing inclusion of populations more representative of those the proposed interventions are intended to benefit. Outside of clinical trials, accurate prognostication could help assess the impact of quality improvement initiatives, training programmes and organisational changes, as well as facilitating interunit comparisons and benchmarking.^40^

## Technical considerations for prognostic tests of biochemical biomarkers for use in resource-limited settings

Markers of common pathophysiological pathways can improve identification of febrile patients with a poor prognosis.^41^ However, the potential for prognostic biomarker tests to contribute to febrile illness management in LMICs is inextricably linked to the human and technical capacity in the settings in which they would be deployed, which in turn defines the technology requirements for test design.

For tests performed at the community level, simplicity is key. Tests will require the most robust features to address stability and transport stress across wide temperature and humidity ranges. Additionally, these tests need to be energy-independent and results must be straightforward to interpret. For prognostic biomarker tests this poses a specific design challenge as many emerging biomarkers are concentration dependent, requiring either quantitative or semiquantitative results.^11^ For improved quantitative assessment, a rapid test reader that can be reliably and affordably used at the community level in LMICs will be required. Reader requirements for use in these settings have been defined but require careful cost–benefit assessment to avoid adding to the biomedical graveyard that already exists in many LMIC primary care contexts.^42^

For tests employed at the district hospital level or above more advanced infrastructure exists (reliable power supply, ambient temperature control, etc) to enable the use of robustly designed instrumented technologies that account for the varied conditions in laboratory settings common in LMICs. Instrumentation permits technologies capable of high sensitivity measurement and quantification, as well as more versatile throughput, sample processing and analyte detection methodologies.

## Future directions

**Figure 3:**
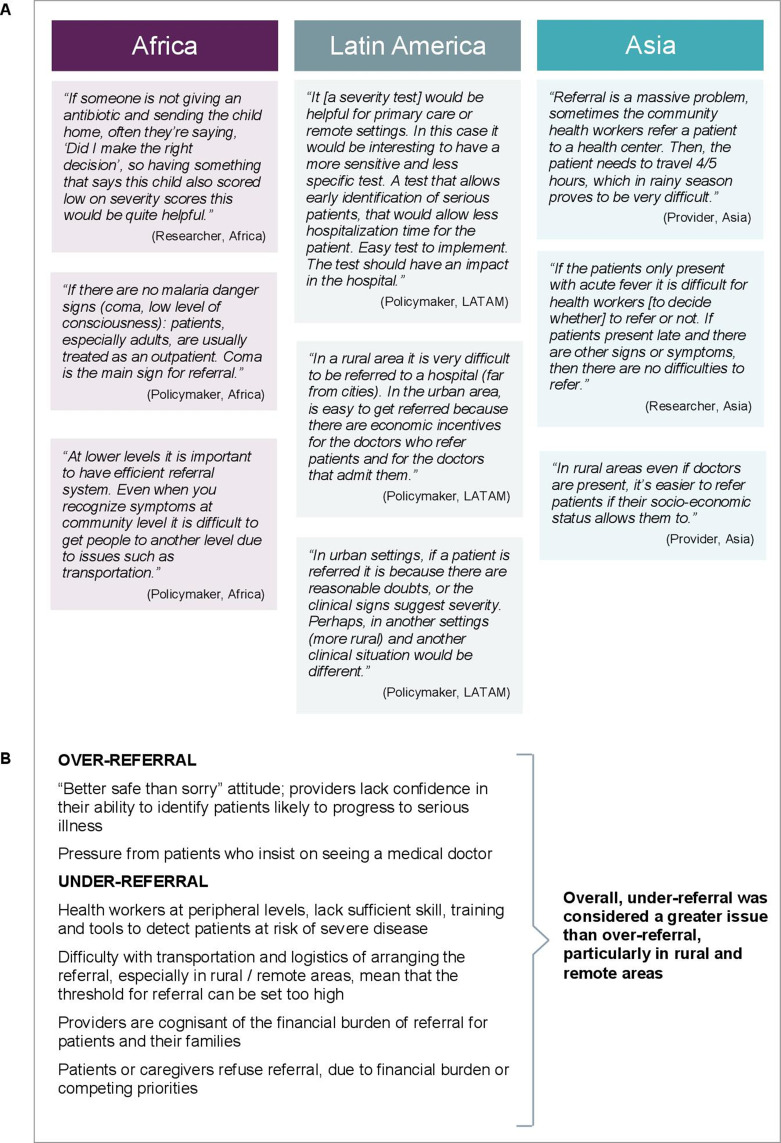
Voices from the field. Opinions of policy makers, healthcare providers and researchers on the opportunities and barriers for prognostic tools in the management of febrile illnesses in heterogeneous resource-limited settings.

Results of a recent multinational stakeholder consultation indicate that better tools to guide community healthcare providers in their referral and admission decisions are urgently required, in particular to prevent under-referral (figure 3).^14^ Crucially, future work evaluating candidate prognostic tests or algorithms for use in community contexts must include individuals managed as outpatients.^43^ Attention must be focused on patients in whom there is clinical uncertainty, rather than on those in whom severe disease can readily be ruled in or out using existing guidelines and algorithms.

The ideal prognostic test or algorithm would have sufficient accuracy and also be generalisable across similar settings. Determining generalisability necessitates comparisons between different studies. Although clearly defined use-cases can harmonise study designs and promote consistency of reporting, standardised assessment methods are essential. Tools to support standardised measurement of baseline predictors have been proposed,^44^ however as the outcome of a febrile illness is influenced by the resources available (a severe outcome may be averted by appropriate treatment) outcome assessment requires a more contextualised approach.

One possibility is to adjust for the differing treatments and interventions a patient receives when data from disparate studies are synthesised. However, treatments and interventions are often applied imprecisely and adjustment for mediating variables is difficult and can introduce selection bias. An alternate solution is to use a standardised tool to assess the overall ‘level of care’ available in different healthcare settings (eg, different treatments, adjunctive procedures and equipment, health worker capacity and provider–patient ratios).^45^ This is already commonplace in critical care medicine, facilitating comparisons across settings with similar ‘levels of care’.^46^ This harmonised yet contextualised approach could be particularly advantageous given the heterogeneous settings in which care is provided for patients living in LMICs.

Additional measures of disease severity, such as need for hospital-level care, must be included alongside mortality. Although surrogate outcomes have important limitations (eg, hospital admission may be influenced by patient and provider preferences), a comprehensive outcome set that includes, but is not limited to these surrogates, is important to consider. Mortality, although a ‘hard’ outcome, occurs infrequently in community settings and predicting death may be of limited utility, compared with predicting severe (and in many instances treatable) illnesses.

Many studies evaluate clinical and biochemical prognostic factors in febrile patients.^47^ Fewer examine the added value of combining biochemical biomarker tests with clinical features. Any new proposed test should add value to the current standard of care. This is particularly important given the added costs and logistical challenges of implementing new tests in decentralised healthcare settings. Many factors determine the optimal sequence for combining different tests, including cost, patient–provider workflow, pretest probability and whether a high NPV or PPV is desired in a particular setting.^48^ Summarising an algorithm’s prognostic capacity using a single metric (eg, the area under the receiver-operating characteristic curve) is of limited use to a health worker confronted with an individual patient.^49^

Few studies consider the additional information that trends in different parameters may provide.^13 50^ Serial measurements of clinical and biochemical parameters may enable more personalised risk prediction than static assessments at a single point in time. To fully realise the benefits of this approach a better understanding of the temporal kinetics of different markers is required.

Finally, once promising prognostic markers are identified, simple, affordable and reliable tests to measure them must be manufactured and supported by robust supply chains to ensure equitable access. Ideally, tests and algorithms would be quantitative, providing a mechanism to adjust cut-offs to achieve the desired NPV or PPV for a particular setting. Candidate predictors already routinely collected for other purposes should be prioritised for evaluation. The growing use of electronic health records may make this more feasible. For clinical features this could include increasing access to technologies such as pulse oximetry and other vital sign devices.^51^ For biochemical biomarker measurements, simple lateral flow tests (with or without quantitative readers), analogous to those in widespread use for the diagnosis of malaria could be envisioned (table 2).

**Table 2:** A practical way forward. Recommendations for researchers, product developers, policy makers and funders to accelerate the development and implementation of prognostic tools for the management of febrile illnesses in resource-limited settings, informed by a recent stakeholder consultation exercise.

Practical steps to improve the design and reporting of studies aiming to accelerate the development and implementation of prognostic tools for the management of febrile illnesses in resource-limited settings			
	Researchers	Product developers	Policy makers and funders
**1. Describe and respect the clinical use-case that the prognostic test or algorithm aims to fulfil** *The study population must reflect the clinical problem that the novel test or algorithm aims to address, for example, the inclusion of outpatients for studies aiming to develop tools for community-based use. Technology must be developed in partnership with users to ensure it meets their needs. Integrated care models must be advocated for and adopted rather than vertical disease-specific programmes, and training of health workers must be prioritised to support the sustained uptake of new tools.*	✓✓✓	✓✓✓	✓✓✓
**2. Measure candidate predictors using common frameworks for data collection** *Candidate predictors should be measured using comparable methodologies to encourage data sharing,**44* *and predictors already identified as promising must be included to allow evaluation of external validity.**47 52 53*	✓✓✓	✓✓	✓✓
**3. Define relevant outcomes against which candidate predictor(s) will be assessed** *Comprehensive outcome sets that include surrogate endpoints must be defined, particularly for use-cases where mortality may not be a relevant or feasible outcome. Ideally these should be prospectively agreed on by all members of the research community.**54*	✓✓✓	✓	✓
**4. Use standardised tools to assess human and material resources available in the targeted settings** *Study settings must be described using standardised tools to contextualise findings and encourage pooling of data from similar environments.**45*	✓✓✓	✓	✓
**5. Report findings in accordance with existing guidelines** *Study design must be adequately reported (eg, the proportion of participants who had met the endpoint at the time candidate predictors were measured)**27* *and results should be summarised using metrics that reflect clinical decision making (eg, positive and negative predictive values, likelihood ratios and net-benefit analyses). Simple technology that can provide quantitative outputs should be invested in to allow cut-offs to be tailored to different risk-benefit scenarios.*	✓✓✓	✓✓✓	✓✓

Number of checkmarks indicate the relative importance of each recommendation for each group.

Prognostic tools that improve risk stratification of patients with febrile illness would have enormous potential to improve patient outcomes and allocation of scarce resources. Each proposed technology requires careful cost-benefit assessment and must be developed in partnership with the healthcare providers working within the targeted contexts. Defining essential product design requirements in consultation with users is essential to ensure usability and promote understanding, acceptance and trust of these technologies. Importantly, donors and implementers must embrace integrated community care and move away from vertical disease-specific models, as the settings where prognostic tools could have greatest impact are precisely the contexts in which diagnosis remains most challenging.

## Data Availability

No data are associated with this work. The findings of the stakeholder consultation conducted by FIND are available at the FIND website: https://www.finddx.org/reports-and-landscapes/meeting-report-biomarkers-for-acute-febrile-illness-at-the-point-of-care-in-low-resource-settings/
